# Near-wall dynamics of single cavitation bubbles imaged by total internal reflection fluorescence

**DOI:** 10.1016/j.ultsonch.2025.107511

**Published:** 2025-08-14

**Authors:** Fangyi Wang, Jonas Kühlmann, Sebastian A. Kaiser

**Affiliations:** Institute of Energy and Material Process – Reactive Fluids, University of Duisburg-Essen, Germany

**Keywords:** Laser-induced cavitation, Total internal reflection fluorescence, Evanescent wave microscopy, Shock wave, Cavitation damage

## Abstract

This study introduces total internal reflection fluorescence (TIRF) as a method for investigating the dynamics of a single laser-induced cavitation bubble in water within a thin layer near a solid surface. TIRF, supported by a planar waveguide, was employed to image cavitation bubble collapses near the liquid–solid interface at 50 thousand frames per second (kfps). Simultaneously, traditional side-view background illumination at 150 kfps captured the bubble dynamics. Dilution series and thickness estimation were conducted to determine interfering fluorescence signal contributions. The results confirm that most fluorescence is excited by the evanescent field rather than surface scattering or bulk scattering. The lifecycle of cavitation bubbles with moderate non-dimensional stand-off distances, γ = 1.15, 1.35, and 1.6, are discussed. We find that a thin liquid film persists between the bubble and the solid surface throughout the entire collapse process, except for randomly distributed micron-sized bubbles attached to the surface between the first and second collapse. Shock waves were captured with TIRF during the second collapse, centered around regions of the toroidal cavity exhibiting locally stronger dynamics. These regions, termed gas-filled strong collapse areas (SCAs), were also where microcracks formed during the second collapse of a single cavitation bubble. While not all SCAs resulted in microcracks, every observed microcrack could be traced back to a preceding SCA. To our knowledge, this is the first application of TIRF to observe cavitation bubble collapses with sub-millisecond time resolution.

## 1 Introduction

Cavitation refers to the formation and subsequent collapse of vapor bubbles in a liquid due to local pressure fluctuations. While the collapse of cavitation bubbles is of great interest in fields such as sonochemistry and its dynamics have been investigated in detail [1], much of the practical relevance of cavitation arises from its interaction with nearby solid surfaces [2]. Near-wall collapse is commonly observed in hydraulic systems, including nozzles, pumps, turbomachinery, and ship propellers, where it can lead to surface pitting[3], material fatigue[4], and efficiency losses accompanied by noise[5]. Despite these detrimental effects, cavitation can also be harnessed for beneficial applications, such as surface cleaning[6], material synthesis[7], medical treatments[8], and wastewater processing[9]. Whether aiming to mitigate its harmful impacts or leverage its potential, understanding the cavitation bubble dynamics near solid surfaces is crucial.

High-speed back-illumination imaging is widely used to investigate cavitation bubble dynamics [10,11]. Lauterborn et al. [12] conducted early studies on cavitation bubble collapse near solid surfaces using high-speed photography. Their research demonstrated that bubbles collapse asymmetrically near boundaries, generating high-velocity microjets directed toward the surface [13,14]. Post-collapse toroidal bubbles were observed undergoing multiple subsequent collapses. Surface damage occurred when the liquid–gas interface of the collapsing bubble was in close proximity to the solid boundary. Shock waves emitted during bubble collapse were detected, indicating a role in cavitation-induced erosion [15].

Most current research focuses on cavitation behavior in bulk liquids, while the detailed near-wall dynamics close to interfaces of cavitation bubbles remain underexplored. You Lung et al.[16] utilized the fringes of equal chromatic order in an optical interference technique to observe, at the submicroscopic level, the rapid formation and collapse of vapor cavities between two moving surfaces and their effects on surface deformation and wear. Several numerical simulation models were proposed to simulate near-wall behavior. For instance, Bremond et al.[17] studied the film thinning process using potential flow boundary integral simulations, and Lauer et al.[18] employed a sharp-interface numerical model to track interface evolution during collapse and rebound. However, the development of relevant models for thin-layer dynamics still lacks consensus, primarily due to the dearth of experimental data on the liquid flow between the vapor cavity and the wall.

More recently, Reuter et al.[19] investigated cavitation bubbles with stand-off distances ranging from 0.47 to 1.07 using the total internal reflection (TIR) shadowmetry. They found that prior to jet impact, each cavitation bubble did not directly contact the solid surface but was separated from it by a liquid film. Using this experimental data, Denner et al.[20] validated their fully coupled pressure-based algorithm and finite-volume framework. Bußmann et al.[21] introduced a sharp-interface level-set method to study the liquid film between the bubble and the wall and validated the model based on that data set. However, the images from TIR shadowmetry are difficult to interpret after the jet impact when the bubble dynamics become more chaotic with strong local interface curvature[19]. It is then not possible to unambiguously determine if the solid surface is locally wetted or not. This is particularly unfortunate because previous research [[22], [23], [24], [25]] shows that for all but the shortest stand-off distances [25], the pit-shaped initial surface damage is mostly attributed to this phase of the collapse. To address this limitation, this work introduces total internal reflection fluorescence (TIRF) to visualize the near-wall dynamics of laser-induced single cavitation bubbles, in particular after the jet impact on the wall.

The concepts underlying TIRF are well established. When light travels from a medium with higher refractive index (RI) $n_{1}$ to a medium with lower RI $n_{2}$ at an angle of incidence greater than a critical angle, all of the light is reflected at the interface – “total internal reflection”. TIR also generates a localized electromagnetic field in the medium with lower RI, known as the evanescent field (EF). For visible light, the EF reaches on the order of 100  nm into the lower-RI medium. It can be utilized to excite fluorescence that is then also limited to this very near-wall region. Typically, the term “TIR fluorescence” (TIRF) is used, even though it is the EF rather than the reflected light that excites the fluorescence.

TIRF is widely used in the life sciences, particularly for studying relatively slow processes, such as cell membrane protein transport [26], single-molecule dynamics [27], and magnetic imaging of living cells [28]. Its applications in engineering also have been somewhat limited to slower processes due to the weak signal associated with the very small probe volume. Chan et al.[29] used TIRF microscopy to study nanobubbles with a temporal resolution of 56  ms. Jin et al.[30] combined TIRF with particle tracking velocimetry to measure the particle slip velocity under relatively low shear rates. TIRF has also demonstrated potential for measuring the near-wall temperature using temperature-sensitive dyes [31].

The technique's good signal-to-background ratio, resulting from the thin illuminated layer, makes it particularly advantageous for near-wall studies. But the transient and fast nature of cavitation bubble dynamics poses significant challenges for implementing TIRF. In this study, TIRF is combined with background illumination (BGI) to investigate the near-wall dynamics of cavitation bubbles. Both techniques are synchronized and applied to laser-induced cavitation bubbles at varying stand-off distances. The near-wall dynamics, shock waves, and microcracks are discussed based on the images captured after jet impact on the solid surface.

## 2 Methods and materials

### 2.1 Total internal reflection fluorescence

**Fig. 1:**
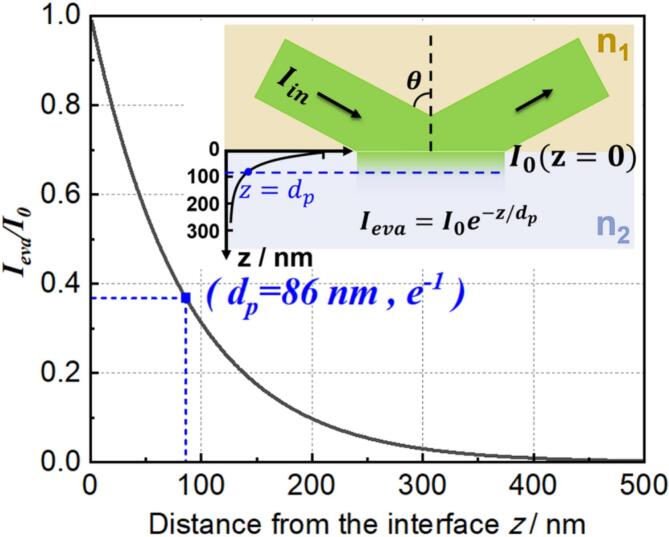
Relative intensity of evanescent field as a function of distance from interface. $n_{1}$ = 1.46, $n_{2}$ = 1.335, λ = 532  nm, θ = 77 ${\;}^{{}^{\circ}}$.

Fig. 1 shows the geometry and properties of TIR. The EF intensity *I*_eva_ decays exponentially with the distance *z* from the interface [32] as given by

(1) $$I_{\mathrm{eva}}=I_{0}e^{-z/d_{p}}$$

where $I_{0}$ is the EF intensity at the wall (z = 0) and *d*_p_ is a characteristic penetration depth (see below). $I_{0}$ is polarization-dependent and depends on the incident light $I_{\mathrm{in}}$, where ${I_{0}}^{\|}$ is $I_{0}$ in p-polarization, ${I_{0}}^{⊥}$ is $I_{0}$ in s-polarization, and $n=\frac{n_{2}}{n_{1}}{(n}_{1}>n_{2})$

(2) $${I_{0}}^{\|}={I_{\mathrm{in}}}^{\|}\frac{4\cos^{2}\theta \left(2\sin^{2}\theta -n^{2}\right)}{n^{4}\cos^{2}\theta +\sin^{2}\theta -n^{2}}$$

(3) $${I_{0}}^{⊥}={I_{\mathrm{in}}}^{⊥}\frac{4\cos^{2}\theta }{1-n^{2}}$$

(4) $$d_{p}=\frac{\lambda_{0}}{4\pi \sqrt{{\left(n_{1}\sin\theta \right)}^{2}-{n_{2}}^{2}}}$$

Fig. 1 displays the normalized EF intensity as a function of distance from the interface. The parameters in the current work are given in the caption and are discussed in section 2.2. The 1/e penetration depth *d*_p_ here is about 86  nm according to eq. (4).

### 2.2 Optical arrangement

**Fig. 2:**
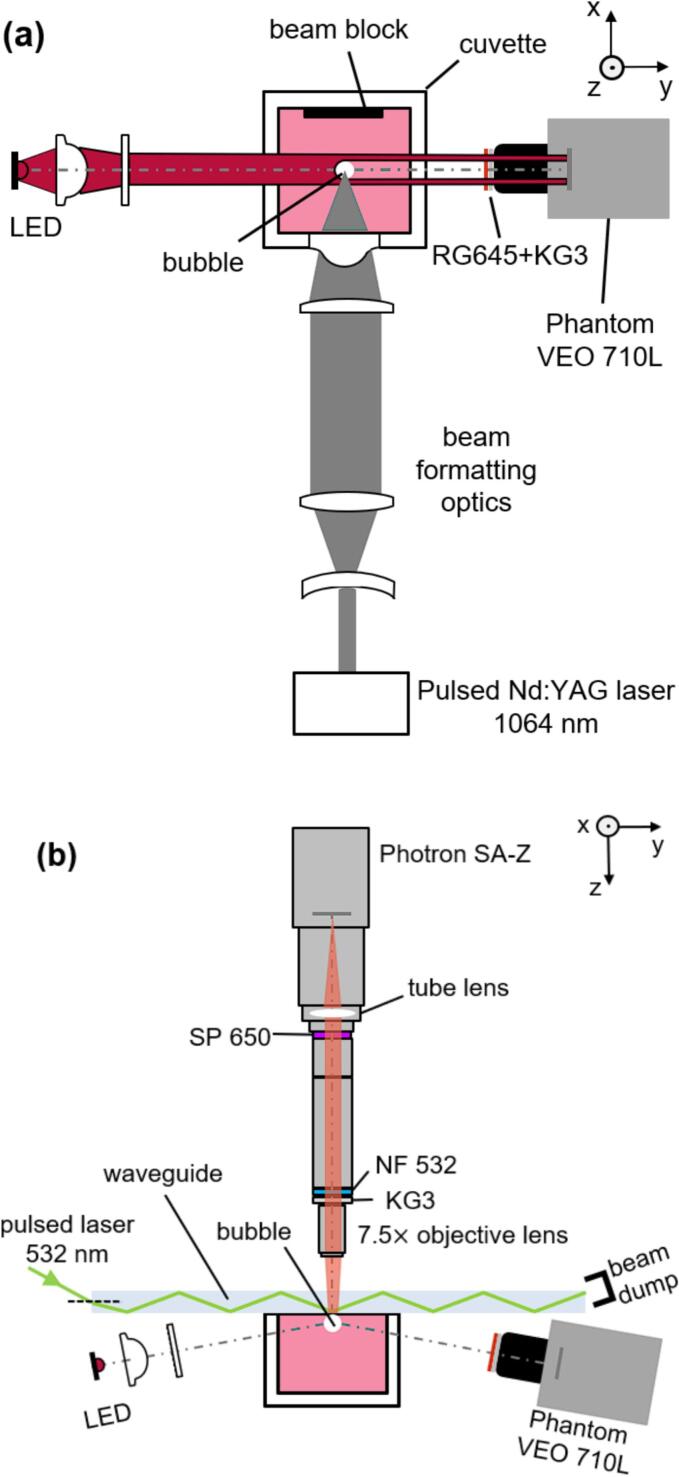
Schematic views of the experiment: (a) top-view without TIRF optics; (b) side view without bubble-generating optics.

**Fig. 3:**
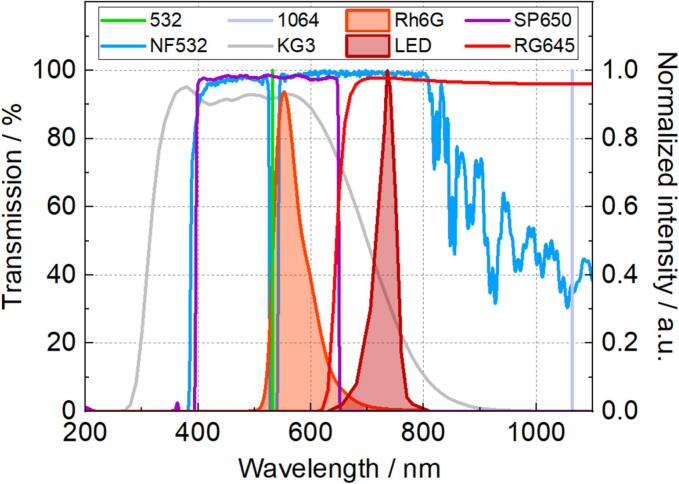
Spectral features of the experiment.

The single cavitation bubble experiment has been described in detail in our previous work [23]. Here, a brief summary is given, and new features are described. Fig. 2 shows the illumination and image detection setup, while the main spectral characteristics are displayed in Fig. 3. The cuvette was filled with rhodamine 6G (Rh6G) diluted in distilled water with a concentration of 0.4  g/L. Cavitation bubbles were generated by focusing the output of a Q-switched Nd:YAG laser at 1064  nm with a repetition rate of 1  Hz. In the side view, the bubble was illuminated by a red LED (centered at 735  nm) and imaged by a macro lens (LAOWA 100  mm f/2.8 2:1) and a high-speed camera (Phantom VEO 710L). At a frame size of 120 ×256 pixels, the camera operated at 150 thousand frames per second (kfps) with a projected pixel size of about 36 × 36 μm in the object plane. Schott KG3 and RG645 filters blocked the laser light at 1064 and 532  nm, respectively. The LED and Phantom camera were positioned at an angle to the horizontal, partly due to space constraints.

A fused silica plate (length × width × height: 200 × 50 × 5 mm, GVB GmbH, Germany) polished on all sides was placed on top of the cuvette and served as a waveguide for the light from a 532  nm laser (Edgewave InnoSlab PS-IS) emitting with 50  kHz repetition rate, a pulse duration of about 10  ns, and an average pulse energy of 0.8 mJ. The laser was incident onto the front side surface of the glass plate to achieve TIR at water–glass interface. A relatively long glass plate was selected to filter out incident rays at less than the critical angle, as well as some stray light through the first few TIRs. The laser light exiting the waveguide was collected by a beam dump. The fluorescence images were captured by a high-speed camera (Photron SA-Z) with an image size of 640 × 600 pixels synchronized with the 532  nm laser at 50 kfps. A microscope objective lens (Mitutoyo Plan Apo 7.5 × ) and a tube lens (Raynox DCR-250) provided a magnification of about 4, yielding a projected pixel size of about 5 × 5 μm in the object plane. A notch filter (NF533-17, Thorlabs) and a KG3 filter suppressed the elastic laser scattering, a short pass filter (FESH0650, Thorlabs) suppressed the LED scattering.

A bubble was produced underneath the polished glass plate every second. Cameras and lasers were synchronized so that the bubble dynamics were recorded by simultaneous BGI and TIRF. The illumination sources were on continuously during each experiment, which usually lasted a few seconds. Both high-speed cameras started recording before the bubble initiation to record baseline images, some of which were used in post-processing, as discussed in section 3.1. A few hundred images are then recorded during the collapse of each cavitation bubble.

### 2.3 Fluorescence signal contributions

In TIRF, the only intended fluorescence is excited by the EF (TIRF). However, potential interfering signals, such as scattering [33], also contribute to the overall measured signal. After preliminary experiments, two other sources of fluorescence excitation are considered here:

i) Bulk scattering induced fluorescence (BSF): light traveling inside the waveguide is scattered, and that scattered light can exit the waveguide and excite fluorescence in the liquid. We examined this by adjusting the laser incidence direction to be parallel to the glass top surface such that bulk scattering occurs but not TIR. The resulting fluorescence images were dark, i.e., BSF cannot be detected here.

ii) Surface scattering induced fluorescence (SSF): at the interface where TIR occurs, light is scattered due to the roughness of the glass surface. This surface-scattered light then enters the solution and excites fluorescence not only in the 100-nm thick region of the evanescent wave, but potentially throughout the bulk of the fluid. We assessed the potential contribution from SSF as follows.

**Fig. 4:**
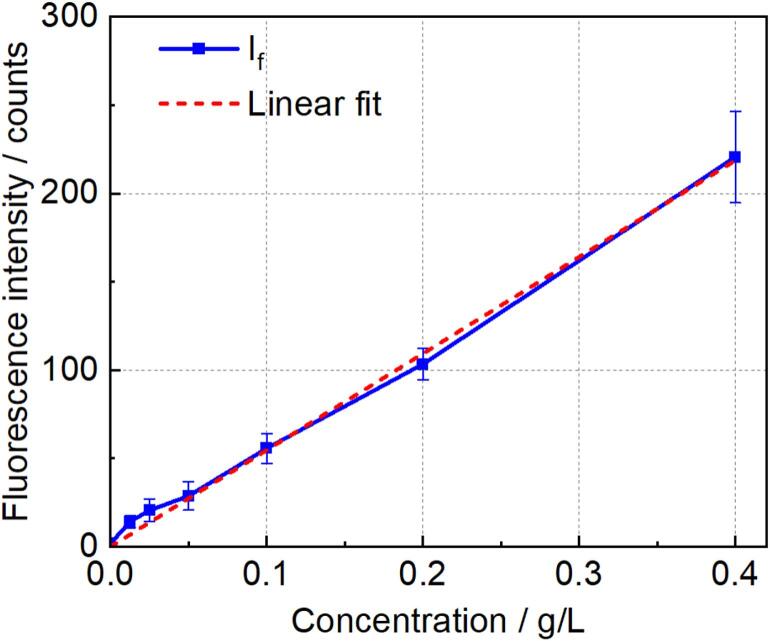
Fluorescence intensity as a function of the Rh6G concentration in water. The error bars represent the variation among eight cycles within one experiment.

Serial dilutions of the fluorescent solution in the concentration range of 0 – 0.4 g/L, with 0  g/L representing pure water, were put into the cuvette (see Fig. 2b). Fig. 4 shows the measured fluorescence intensity as a function of concentration. The square datapoints align well with a linear fit, as illustrated by the dashed line in Fig. 4. The fluorescence signal is approximately 1.75 counts for a concentration of 0  g/L, representing the camera’s dark signal. Since according to eq. (3) the penetration depth of the EF remains constant when varying the concentration, the TIRF intensity should increase (for low enough concentrations, linearly) with dye concentration. In contrast, because the camera captures a signal integrated along the Z-axis throughout the solution, and except for the case of pure water, the solution was sufficiently concentrated to absorb all incident light, the SSF intensity is expected to remain constant for non-zero concentration. Therefore, SSF contributes equally to the fluorescence intensity for all dilutions, represented by the y-intercept in Fig. 4. However, compared to the fluorescence signal at a concentration of 0.4  g/L, the y-intercept is small enough to be considered negligible, suggesting that the interference of SSF on TIRF is minimal.

**Fig. 5:**
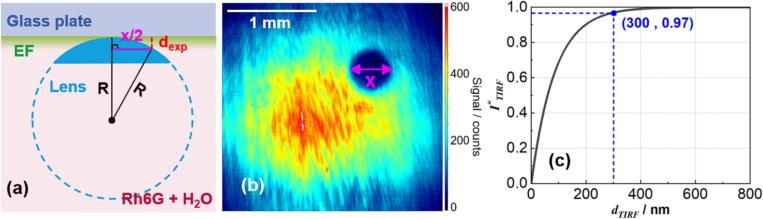
(a) schematic drawing of the optical geometry, (b) local signal depression in the raw TIRF image caused by the lens displacing the fluorescent solution, (c) theoretical normalized depth-integrated TIRF signal ${I^{*}}_{\mathrm{TIRF}}$ as a function of the free space $d_{\mathrm{TIRF}}$ in the solution.

We also estimated the depth to which the dye is illuminated following the method proposed by Mattheyses et al. [33]. A plano-convex lens (f = +200  mm, Ø1″, Thorlabs) with a curvature radius R = 103  mm was immersed in the solution mounted on an adjustable z-axis translation stage. The translation stage was adjusted to lightly push the convex surface of the lens against the waveguide. The optical geometry is illustrated schematically in Fig. 5a. At a distance x/2 from the center, the fluorescence signal stems from the liquid between waveguide and lens with depth $d_{\exp}$ as indicated in Fig. 5a. From trigonometry,

(5) $$d_{\exp}=R-\sqrt{R^{2}-{\left(\frac{x}{2}\right)}^{2}}$$

A raw TIRF image is shown in Fig. 5b. We find that x≈ 495 μm, yielding $d_{\exp}\;\approx$ 300  nm.

Considering TIRF, the normalized depth-integrated TIRF signal ${I^{*}}_{\mathrm{TIRF}}$ as a function of the liquid depth $d_{\mathrm{TIRF}}$ that signal is generated from is given by:

(6) $${I^{*}}_{\mathrm{TIRF}}=\frac{\int_{0}^{d_{\mathrm{TIRF}}}I_{0}e^{-\frac{z}{d_{p}}}\;dz}{\int_{0}^{\infty }I_{0}e^{-\frac{z}{d_{p}}}\;dz}=1-e^{-\frac{d_{\mathrm{TIRF}}}{d_{p}}}$$

Fig. 5c presents the theoretical curve derived from eq. (6) and from eq. (4) for our experiment *d*_p_ = 86  nm.

Considering SSF, the 1/e penetration depth $d_{\mathrm{SSF}}$ of surface scattering in the light-absorbing dye solution is estimated based on the Beer-Lambert law:

(7) $$d_{\mathrm{SSF}}=\frac{1}{\ln10\;∙\;\varepsilon \;∙\;c}\;\approx \;104\;\mu m$$

which is four orders of magnitude more than the EF depth. Here, the extinction coefficient ε is taken as 5 × 10^4^ L/(mol∙cm) [34]. The concentration c is 8.35 × 10^-4^ mol/L(that is, 0.4  g/L).

The measured $d_{\exp}$ is consistent with the predictions based on TIRF theory, represented in Fig. 5c, if ${I^{*}}_{\mathrm{TIRF}}$ = 0.97 $∙\;I_{0}$. Thus, $d_{\exp}$ does not appear to be determined by surface scattering, as $d_{\exp}\;\ll \;d_{\mathrm{SSF}}$. In conclusion, both the dilution test and the lens test indicate that most of the fluorescence signal originates from EF, with SSF and BSF making negligible contributions.

## 3 Results and discussion

Prior to discussing the results, some clarification of nomenclature may be helpful. Throughout the bubble's lifecycle, multiple collapses and rebounds occur[15,35]. In this work, a “collapse” is the contraction of the gas phase within the fluid to a local spatio-temporal minimum. However, if it is not explicitly stated which of the individual spatio-temporal minima is meant (first, second), “collapse” may also refer to the overall process.

### 3.1 Visualization of liquid film between cavitation bubble and solid surface

**Fig. 6:**
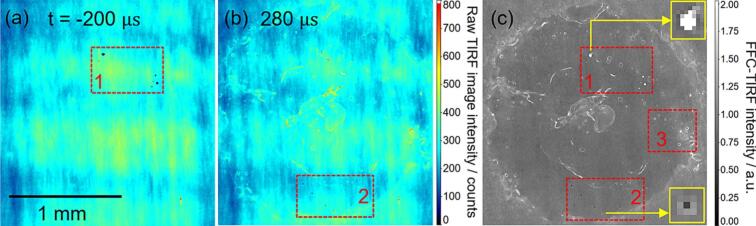
In the same image cycle, (a) a selected raw TIRF image before cavitation bubble generation, (b) a selected raw TIRF image after the first collapse of the cavitation bubble, and (c) image (b) after flat-field correction. Time zero is defined here and elsewhere (unless otherwise mentioned) as the moment when the bubble reaches its maximum diameter. The corresponding video is available in the supplementary materials.

The camera begins recording before each bubble generation and continues throughout the bubble lifecycle. A raw TIRF image is shown in Fig. 6a. The fluorescence distribution is quite non-uniform, which is due to the uneven transverse beam profile. An average over 75 such images from before the cavitation bubble is generated (here, t = 200 μs), serves as a reference (a “flat field”) for the subsequent cavitation bubble images. In Fig. 6a, the red box labeled “1” contains small “black” spots with low fluorescence signal. These are likely gas bubbles already attached to the waveguide surface, as the gas phase is non-fluorescent.

From the same cycle, Fig. 6b shows a raw TIRF image after a cavitation bubble had been generated and underwent its first collapse. Red box 2 contains small dissolved-gas bubbles similar to those in box 1. Nevertheless, the large-scale pattern of the fluorescence signal in Fig. 6b is quite similar to that in Fig. 6a and is dominated by the footprint of the transverse beam profile. To remove this laser inhomogeneity, a flat-field corrected (FFC) image $I_{\mathrm{TIRF}}^{*}$ was calculated according to:

(8) $$I_{\mathrm{TIRF}\;}^{*}=\frac{I_{\mathrm{TIRF}}-\langle I_{\mathrm{dark}}\rangle }{\langle I_{\mathrm{TIRF}}\rangle -\langle I_{\mathrm{dark}}\rangle }\;$$

where the $I_{\mathrm{TIRF}}$ is the raw TIRF image of the cavitation bubble, $\langle I_{\mathrm{TIRF}}\rangle$ represents the time-averaged flat-field before cavitation bubble generation in the same cycle, and $\langle I_{\mathrm{dark}}\rangle$ is an averaged dark image without fluorescence (usually, simply with the laser turned off, but we also checked that the signal with pure water was nearly that with the laser off).

Fig. 6c presents the flat-field corrected image from Fig. 6b. The positions of the red boxes remain unchanged, with magnified views of even smaller details provided in the inset. Flat-field correction assigns a value close to 1 to represent fluorescent aqueous solution, and a value close to 0 corresponds to the attached gas phase. The attached bubbles already present in the flat field (box 1) now appear as white spots, whereas the attached bubbles after the first collapse (box 2) appear as black spots.

With the stationary image inhomogeneities now removed by flat-fielding, Fig. 6c reveals the near-wall structure of the cavitation bubble after its first collapse. The bubble splashes radially along the waveguide surface, forming a roughly annular (torus) shape. Some small bubbles not attached to the waveguide surface are observed, such as in box 3. The outer radial boundaries of both the cavitation bubble torus and these small bubbles exhibit higher fluorescence intensity than the flat-field image, i.e., values exceeding 1. These could stem from scattering of the EF or scattering of the fluorescence signal by gas–liquid interfaces. That RI discontinuities in the EF cause local scattering was reported in other studies in the context of biology [36,37]. However, Fig. 6c shows that the wall-attached bubbles in box 2 do not have significantly brighter edges than the bubbles away from the wall in box 3. Therefore, it is more likely that it is the fluorescence light that interacts with gas–liquid interfaces, where it is refracted and may even undergo local TIR because of the curvature of the interface. This effect may depend on the smoothness of the interface as well as the number of interface folds. Therefore, these areas, as well as the thin white curves in Fig. 6c, are assigned to gas–liquid interfaces that are close to the wall but not necessarily in the EF, with more spatially distributed high-signal areas corresponding to aggregations of corrugated interfaces.

Among the several hundred cavitation bubbles examined in this work at stand-off distances (γ) ranging from 1.0 to 1.7, attached micron-sized bubbles (such as in box 2) were observed only between the first and second collapse. Their spatial distribution was relatively random, and their motion appears to be driven by local pressure changes caused by the liquid flow around the cavitation bubble, as discussed below.

### 3.2 Near-wall dynamics of bubbles with different stand-off distances

Considering the different frame rates of BGI (150  kHz) and TIRF (50  kHz), we aligned the base frame of both methods to the one capturing the second collapse of the cavitation bubble. For comparison with the corresponding TIRF images the BGI sequences were then subsampled by a factor of 3.

**Fig. 7:**
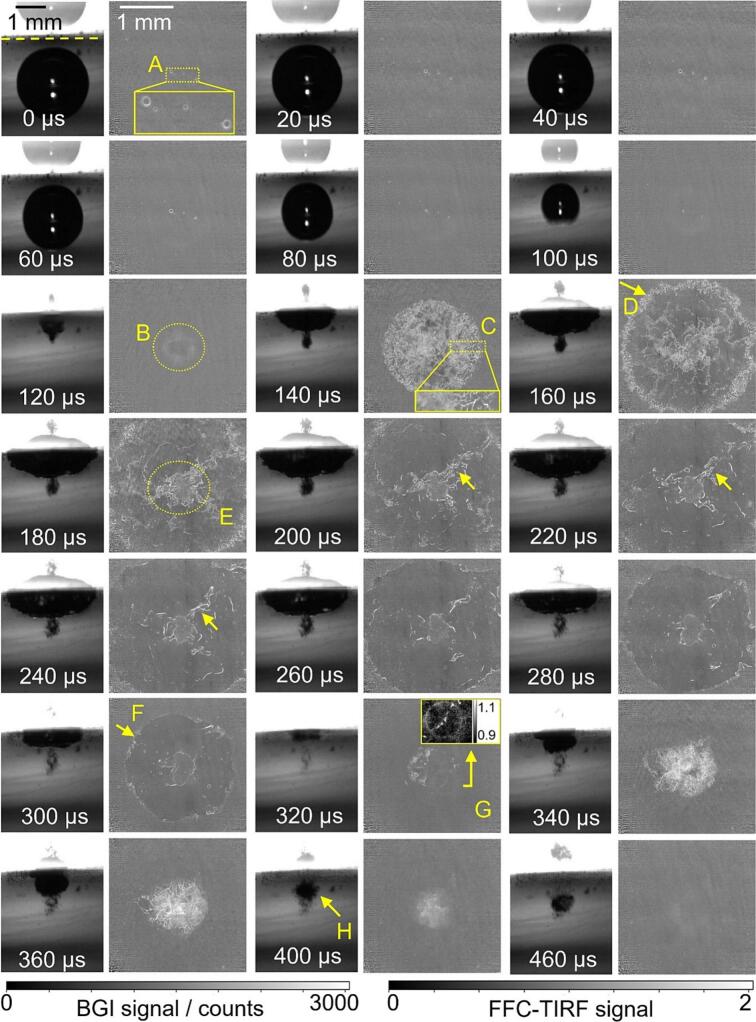
BGI images (left) and TIRF images (right) of a cavitation bubble with r = 1.2  mm, γ = 1.15. Letter labels are discussed in the text. Note that the two image types (BGI vs TIRF) are shown at different magnifications, as indicated by the scale bars. The corresponding videos are available in the supplementary materials.

Fig. 7 shows an image sequence of the lifecycle of a cavitation bubble withγ = 1.15 and r = 1.2 mm. The laser beam generating the cavitation bubbles was incident from the left. In the BGI images, the lower section shows the bubble’s shadow, while the upper section displays the specular reflection of the bubble on the glass plate (waveguide). The yellow dashed line in the first BGI image of Fig. 7 marks the symmetry line, i.e., the location of the solid surface in the focal plane, used to calculate γ.^1^ The time when the cavitation bubble diameter reaches its maximum in BGI is set as 0 μs. Several interesting phenomena are discussed in chronological order, corresponding to the letters marked in Fig. 7.

A. At t = 0  μs, apart from the large cavitation bubble, there are several small bubbles distributed along the symmetry line in the BGI image. These bubbles are remnants from the previous bubble cycle. Correspondingly, the TIRF image also shows some of these bubbles with bright edges. However, the near-unity fluorescence signal in the bubble centers indicates that they are not attached to the waveguide but are very close to it. These pre-existing microbubbles undergo expansion (from 0  μs to 60  μs) and contraction (from 60  μs to 100  μs) as shown in TIRF images. The size changes are a response to the pressure variations associated with the bubble collapse. The bubbles also move laterally toward the center of the image (seen much more clearly in the videos provided in the Supplementary Material). This is consistent with the inward flow between the bubble and surface (“microconvection” [38]) driven by the same low-pressure region that causes the bubble to collapse towards the surface.
B. The first collapse occurs between 113  μs and 120  μs, as indicated by the corresponding BGI image sequence in the supplementary materials. At 120  μs, the bubble moves toward the waveguide located in the upper part of the BGI image, while a slightly oval ring-like shape appears in the TIRF image. The signal in the middle of the ring is close to 1, suggesting that it originates from the central liquid jet that is expected at this stage of the collapse [24,38]. The slightly asymmetric shape of the ring may be related to the asymmetry of the initial plasma shape.
C. After the central jet impacts the waveguide, the bubble splashes radially outward at 140  μs, as shown in the BGI image. In the TIRF image, the outer border appears symmetrical, but the interior exhibits irregularly arranged corrugated bright lines with signal levels significantly above 1. These lines are taken to be gas–liquid interfaces. The multitude of corrugated shapes, stemming from instabilities, is consistent with the near total opacity of the bubble seen even in BGI imaging that is much more optimized than in our implementation [[38], [39], [40]]. The magnified inset shows that the lines are “sharper” radially away from the center of the bubble, indicating that the outer portion is in the focal plane, while the central part is farther from the waveguide surface.
D. The bubble continues expanding along the waveguide surface as the BGI images show after 140  μs. The edge consists of many short, curved bright lines, i.e., gas–liquid interfaces, in the TIRF image at 160  μs.
E. The circle marked with ‘E’ in the TIRF image at 180  μs most likely originates from the central jet. The bubble transforms into a torus. Due to shear forces along the wall, thin and long gas lamellae form between the edge and the central jet, as indicated by the arrows from 200  μs to 240  μs. A similar “inverted-foam” topology was reported by Reuter et al [19].
F. The BGI images show the bubble shrinking inward from 240  μs to 320  μs. By 300  μs, the outer edge appears smoother compared to the earlier stage at 160  μs marked with ‘D’. The signal at the outer front marked with ‘F’ is also much lower than at ‘D’, consistent with the idea that the camera-detected fluorescence signal is influenced by the curvature of nearby gas–liquid interfaces. Both BGI and TIRF indicate that the outer edge of the bubble contracts significantly inward, while TIRF also reveals that the edge dimensions of the central jet flow remain relatively unchanged.
G. At 320  μs, the toroidal bubble reaches its minimum volume, marking the second collapse. Part of the bubble is visible on the left side of the TIRF image. At the 5 o’clock position, a nearly perfect circle with an intensity slightly above 1 is observed – see the inset for better contrast. We interpret this circle to represent a shock wave. It will be discussed in more detail later.
H. After the second collapse, the gas cavity moves away from the surface [15], as shown in the BGI images from 340  μs to 460  μs. The elevated TIRF signal in the image center at 400  μs is attributed to the presence of many small bubbles that create RI discontinuities, leading to scattering of the evanescent wave [36,37] and the fluorescence signal. The bubble marked with ‘H’ seems more like a foam or dense cluster of many microbubbles than a contiguous gas-filled cavity [15]. Finally, the TIRF signal approaches that of the pre-bubble reference images again, i.e., unity in the corrected images shown at 460 μs.

**Fig. 8:**
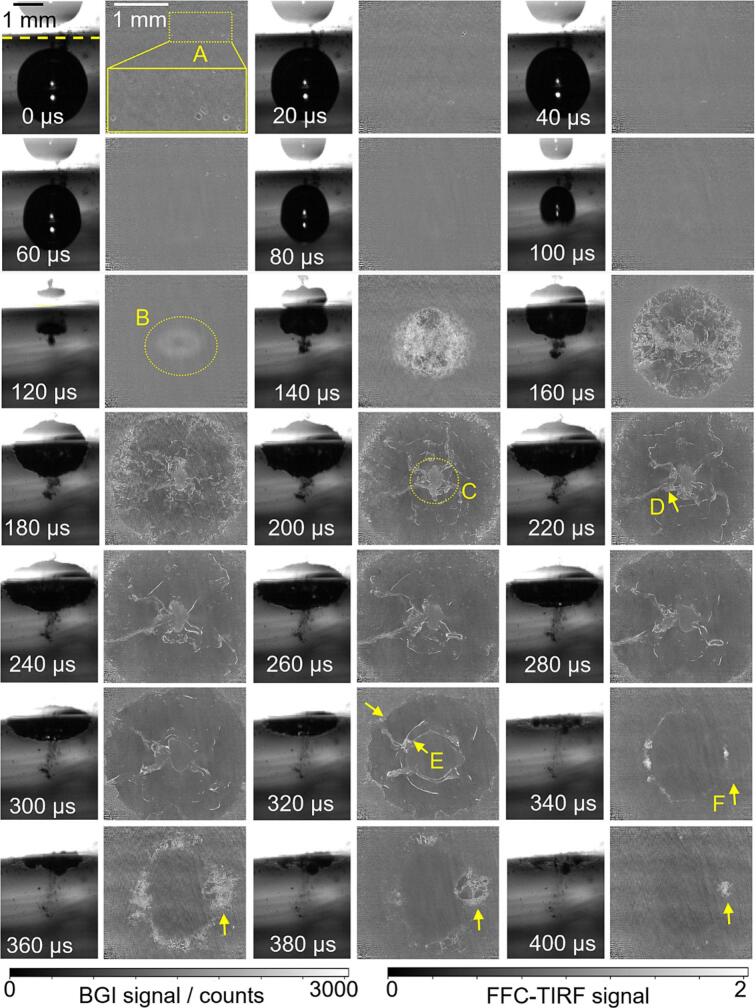
BGI images (left) and TIRF images (right) of a cavitation bubble with r = 1.3  mm, γ= 1.35. The corresponding videos are available in the supplementary materials.

Results forγ = 1.35 are displayed in Fig. 8. Box ‘A’ marks pre-existing microbubbles similar to those in Fig. 7. From 0  μs to 100  μs these bubbles expand and contract in response to the pressure created by the main bubble. The first collapse occurs between 113  μs and 120  μs, as indicated by the corresponding BGI image sequence. At 120  μs, the oval ring-shaped fluorescence signal marked with ‘B’ indicates that the central jet has pierced through the bubble after the first collapse. From 140  μs to 240  μs, the bubble spreads outward along the waveguide surface. ‘C’ marks a region with near-unity signal in bubble center in the TIRF image at 200  μs, which appears to originate from the liquid jet. Irregularly shaped, elongated protrusions (indicated by ‘D’) are observed in TIRF images and can be tracked from 220  μs to 320  μs. They may represent the lamellae structures seen in our earlier work [19]. ‘E’ marks the folding out of the inner boundary and the contraction of the outer boundary in the TIRF image at 320  μs. A smooth, very thin bright semi-circle is seen between the inner and outer edges. Then the second collapse occurs at 340  μs, with ‘F’ marking a shock wave. The TIRF image at 360  μs exhibits a high-frequency stripe pattern, which is attributed to shock wave-induced waveguide vibrations. They result in spatial misalignment between the flat-field reference image and the raw TIRF image, creating the pattern upon division.

In our previous work[24], shock waves were captured in BGI with relatively short LED pulse illumination (about 200  ns), but to obtain sharp images of the shock wave in water, nanosecond [41,42] or sub-picosecond [25] illumination or exposure time is needed. Given the 10  ns laser pulse and that the fluorescence lifetime of Rh6G is only a few nanoseconds, it is reasonable that shock waves appear as sharp features in the TIRF images here. Each time a cavitation bubble collapses, it may emit several shock waves. However, among the hundreds of cavitation bubbles tested in this study (with γ ranging from 1 to 1.7), only during the second collapse shock waves were seen. A shock wave from the first collapse was not observed, even though it may be expected to contain higher initial energy. This may be because the first collapse occurs farther from the waveguide surface compared to the second collapse, causing the shock wave to weaken by the time it reaches the EF. Additionally, the second collapse is more asymmetric, while the toroidal bubble stage collapses asynchronously, generating multiple shock waves and thereby increasing the likelihood of capturing one with the relatively slow 50 kfps frame rate [43].

The shock wave in Fig. 8 is centered around locally brighter spots within the bubble torus. We infer that the increased brightness in these regions is due to the density or particular distribution of gas–liquid interfaces. These spots enlarge and evolve as more gas–liquid interfaces fill the expanding regions from 360  μs to 400  μs in the TIRF images of Fig. 8. This behavior was previously referred to as locally enlarged ('strong') gas-filled collapse areas (SCAs) in our earlier study [23], and we use the same term here for consistency. In Fig. 8, the shock wave originates from the SCA located at the 3o’clock position. The energy per unit area of a spherically propagating shock wave decreases with the square of the distance from the origin, indicating that the local pressures in these SCAs would be even higher than at the moment when the TIRF imaging captured the shock wave.

**Fig. 9:**
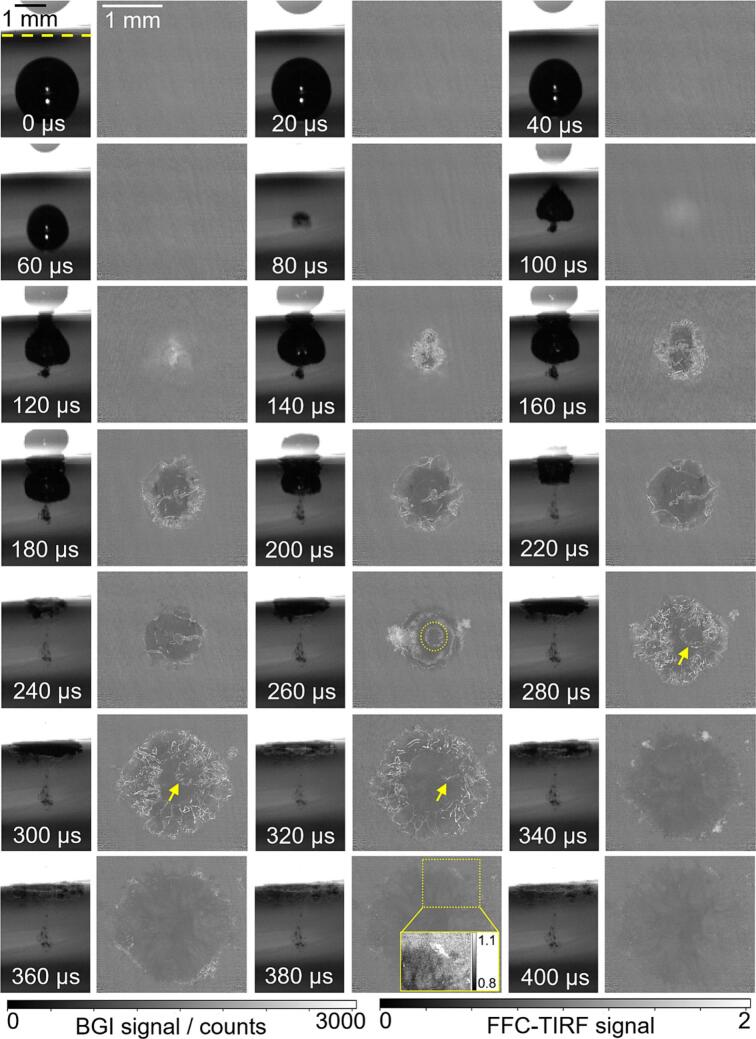
BGI images (left) and TIRF images (right) of a cavitation bubble with r = 1  mm, γ = 1.6. The corresponding videos are available in the supplementary materials.

Images for γ = 1.6 are shown in Fig. 9. This was the first bubble in a series, so no microbubbles are present. In addition to what was seen for the smaller stand-off distances, two new features are observed here or can be seen more clearly than in the previous examples. First, when the second collapse occurs between 240  μs and 260  μs, two concentric collapse tori are observed at 260  μs in TIRF image. The inner torus is marked with a dashed circle at 260  μs, and it moves towards the 2o’ clock position in the subsequent frames, presumably due to bubble asymmetry. This double torus structure resembles the multi-bubble damage patterns on ductile materials reported in previous research – a ring plus central damage for γ = 1.69 and 1.52 [15] and 1.55 to 1.9 [24]. Second, during bubble collapse from 160  μs to 320  μs, the FF-corrected signal in an extended area inside the bubble edge is lower than 1 in the TIRF images. Typical values at 200  μs are 0.75. The sub-unity signal could mean that the liquid layer is thinner than the depth of the EF here, with Fig. 5c then indicating a film thickness of about 120  nm. At 320  μs, following the third collapse, the toroidal bubble transforms into a ring-shaped cluster of microbubbles [40] that are approximately evenly distributed along the circumference.

After the second collapse, from 340  μs to 400  μs radial striation (see inset at 380  μs) appears without the sharp brighter lines that we attribute to gas/liquid interfaces, indicating that liquid/liquid convection may play a role. We think this phenomenon is related to temperature variations in the liquid. Fluorescence of Rh6G is temperature-sensitive. In water at about 300  K, the fluorescence yield increases by about 20 % for a temperature increase of 10  K [44]. In fact, high-speed fluorescence microscopy has been used to analyze temperature changes associated with micrometer-sized cavitation bubbles [45]. However, in our case, we think the observed temperature change is only indirectly associated with the bubble. The laser runs for several seconds before the bubble, and absorption by the dye may heat the liquid within the EF. The flow associated with the bubble collapse then brings cooler liquid from the bulk into the boundary layer. To test this hypothesis, we performed the same experiment with Rhodamine B (RhB) as a dye. Compared to Rh6G, RhB exhibits the opposite fluorescence intensity dependence on temperature [44,46]. We found that indeed, with RhB the FF-corrected signal in the corresponding regions after the second collapse was above 1, confirming our conclusion that the darker regions in Fig. 9 and the striation at late times represent convection and mixing of cooler bulk liquid into the laser-heated wall boundary layer.

**Fig. 10:**
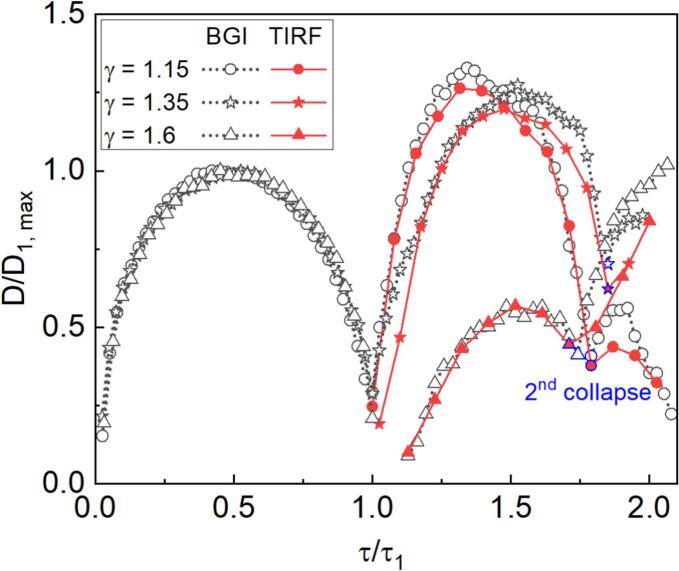
Comparison of the bubble diameter between BGI and TIRF images with γ= 1.15, 1.35, 1.6.

To examine the consistency between the two imaging methods, we compare the apparent size of the bubble in BGI and TIRF. During the initial growth and (first) collapse, the bubble is only visible in BGI imaging because it is not close to the wall. In this phase, the diameter was determined by fitting a hemisphere to the portion of the bubble facing away from the wall, as for example in [19]. This allows determining the collapse time $\tau_{1}$ and the maximum diameter $D_{1,max}$ (in analogy with completely spherical bubbles). For times after $\tau_{1}$, in the BGI images the diameter of the now toroidal bubble is estimated by identifying the laterally outermost shadow near the wall. From TIRF the bubble diameter is extracted by fitting a circle to the outermost bright interface lines. Fig. 10 shows the resulting plot, in coordinates normalized to account for the slightly different bubble sizes. The discontinuity in the BGI-derived trace for γ= 1.6 corresponds to the time interval between the first collapse and the subsequent impact on the wall. Between the first and second collapse, the diameters obtained from TIRF and BGI show good agreement, indicating consistency between the two imaging methods. The bubble undergoes its second expansion–contraction cycle during this stage. The diameter near the second collapse is marked in blue for each γ. Following the second collapse, lateral expansion is observed again, but the differences between what is observed with the two methods are now greater.

Now that we have examined the results for a range of stand-off distances, a couple of broader observations can be made. First, for all γ discussed in Fig. 7, Fig. 8, Fig. 9, the BGI image sequences are consistent with the literature on single laser-generated bubbles in pure water [14,15,47]. This suggests that the dye has little effect on the overall bubble dynamics, despite its potential influence via viscosity and surface tension. Second, the TIRF images show that apart from microbubbles attached to the waveguide surface between the first and second collapse (for example, see Fig. 6c), we *never* see signals approaching zero that would indicate a locally dry solid surface. Therefore, a liquid film thicker than at least 120  nm is *always* present between the cavitation bubble and the glass surface throughout the entire bubble lifecycle.

### 3.3 Cavitation damage − microcracks

Our previous work[[22], [23], [24]] focused on cavitation-induced damage on Cu-, Fe-, and Al-based alloys, for which we found that damage pits could form after a single cavitation bubble collapse, due to the second collapse. Similarly, in this study using a much more brittle fused silica plate [48], microcracks were observed following the collapse of a single cavitation bubble.

**Fig. 11:**
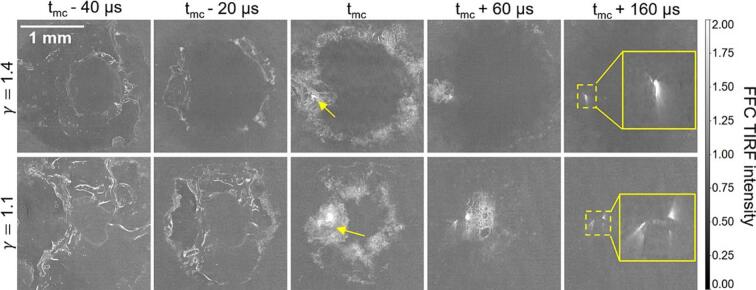
Generation and evolution of damage on the waveguide surface during the second collapse for two different stand-off distances. Image timings are given with respect to the first appearance of what is interpreted as a microcrack ($t_{mc}$).

In Fig. 11, the first row shows the time series of TIRF images before and after the second collapse of a bubble with γ = 1.4 and in the second row with γ = 1.1. The second collapse occurs between the t_mc_ - 20  μs and t_mc_ for both γ. We interpret the bright spots observed at t_mc_ to be the first appearance of microcracks. The insets at t_mc_ + 160  μs show sharp fluorescence signal structures outwardly radiating from the microcracks. The damage alters the local geometry, preventing the incident angle from satisfying the critical angle. The laser light, incident from the top, is refracted into the fluorescent solution, generating fluorescence intensity several times higher than that in FF image.

The high-signal signatures of pronounced SCAs are observed at t_mc_ + 60  μs. They originate from the local asymmetry of the second collapse. The SCAs are located at the 3o’clock position for both γ, and spatially overlap with the microcracks. We did not observe any other phenomena that could potentially contribute to the damage. Consistent with our previous findings concerning pits on metal samples, not all SCAs resulted in TIRF-detected microcracks, but microcracks initiation was always spatio-temporally associated with SCAs.

## 4 Conclusions

This work introduces total internal reflection fluorescence (TIRF) as a method for observing the near-wall dynamics of cavitation bubbles. To our knowledge, this is the first application of TIRF to visualize the collapse of near-wall cavitation bubbles, or any other near-wall flow, at a sub-millisecond scale (here, with 50 kfps imaging). The study was motivated by our earlier work in which we were able to quantitatively measure the thickness of the liquid film between bubble and solid wall *before* the first collapse but could not conclude whether the wall was locally dry *after* the first collapse. We initially expected to find nearly uniform fluorescence signal, potentially with short-lived dark regions that would then indicate local direct contact of gas phase and wall. However, the images showed a plethora of complex features. For a better interpretation of the TIRF images, serial dilutions and evanescent-field (EF) thickness estimates were conducted. The results demonstrate that most of the fluorescence signal originates from evanescent-field excitation, with minimal contributions from excitation by bulk or surface scattering.

The study demonstrates the feasibility of using TIRF to capture the collapse morphology of laser-induced single cavitation bubbles. The commonly used background illumination was also applied synchronously to capture the bubble dynamics, serving as a benchmark for orientation by comparison with the literature. Hundreds of cavitation bubbles were examined at various non-dimensional stand-off distances (γ) from 1.0 to 1.7 and bubble radii of about 1 to 1.5  mm. The discussion focused on example TIRF image series at γ = 1.15, 1.35, and 1.6. We find that apart from randomly distributed micron-sized bubbles that are sometimes fully attached to the waveguide (glass plate) between the first and second collapse, during its entire “lifecycle” a thin liquid film separates the cavitation bubble from the glass surface. Calculations of the EF thickness from theory indicate that the film is thicker than 120  nm.

TIRF imaging appears to show the gas–liquid interface of the bubble near the solid wall following its impact. Subsequently, the interior of the toroidal bubble boundary becomes filled with curved, thin gas lamellae. As γ increases from 1.15 to 1.6, the location of the second collapse shifts spatially from the inner boundary towards the outer boundary of the then toroidal bubble. The high signal intensity regions observed after the second collapse, resulting from many closely spaced gas–liquid interfaces, indicate strong collapse areas (SCAs) adjacent to the wall. Shock waves were observed at the second collapse of the cavitation bubble, centered around SCAs. These SCAs, resulting from the asymmetric collapse of the cavitation bubble, also showed a strong correlation with the locations of surface microcracks appearing on the waveguide during an image sequence. These findings are consistent with our previous observations of damage pits on ductile materials (metals) [22,23], confirming that for the stand-off distances examined here, regions where SCAs occur during the second collapse are where damage is most likely. Consistent with our previous work, the results here also suggest that shock wave emission (during the second collapse for γ ranging from 1.0 to 1.7) is the dominant mechanism driving damage in single-bubble cavitation.

TIRF primarily creates signal in the evanescent field region, to about 100 – 200 nm from the wall. This provides exceptional sensitivity to near-surface phenomena, but stemming from so little fluid, the signal is relatively weak. The 50  kHz repetition rate used in this study is insufficient to capture all dynamics associated with bubble collapse. Higher frame rates are currently limited more by the available high-power lasers than by cameras. While the fluorescence signal *originates* from the evanescent field, it can be influenced by features (e.g., reflections and refractions) *outside* of that thin layer, such as gas/liquid interfaces. Thereby further features can be seen, but physical interpretation is somewhat complicated. Away from such disturbances, flat-field referencing made our images semi-quantitative in its dependence on the depth-integrated illuminated fluid volume. The local temperature also influences the signal. Finally, TIRF requires a transparent substrate, limiting investigations into surface damage mechanisms. These limitations should be carefully considered when interpreting TIRF-based measurements of cavitation phenomena near solid boundaries.

## CRediT authorship contribution statement

**Fangyi Wang:** Writing – original draft, Visualization, Software, Methodology, Investigation, Formal analysis, Data curation. **Jonas Kühlmann:** Writing – review & editing, Validation. **Sebastian A. Kaiser:** Writing – review & editing, Validation, Supervision, Methodology, Funding acquisition, Conceptualization.

## Declaration of competing interest

The authors declare that they have no known competing financial interests or personal relationships that could have appeared to influence the work reported in this paper.

## Footnotes

1 The location of the solid surface was inferred from the mirror symmetry of the images, which can be seen much better when adjusting the greyscale for that purpose. Compared to some of the literature, e.g. [15][38], the BGI images here are of lower quality because of the greater-than-ideal angle of illumination and camera with respect to the plate, which was mainly imposed by space constraints.
